# ERK1/2 is activated in non-small-cell lung cancer and associated with advanced tumours

**DOI:** 10.1038/sj.bjc.6601644

**Published:** 2004-03-02

**Authors:** S Vicent, J M López-Picazo, G Toledo, M D Lozano, W Torre, C Garcia-Corchón, C Quero, J-C Soria, S Martín-Algarra, R G Manzano, L M Montuenga

**Affiliations:** 1 Carcinogenesis Unit, Division of Oncology, Centre for Applied Medical Research (CIMA), University of Navarra, Pamplona 31008, Spain; 2 Department of Histology and Pathology, University of Navarra, Pamplona 31008, Spain; 3 Department of Oncology, University Hospital, University of Navarra, Pamplona 31008, Spain; 4 Department of Pathology, University Hospital, University of Navarra, Pamplona 31008, Spain; 5 Department of Thoracic Surgery, University Hospital, University of Navarra, Pamplona 31008, Spain; 6 Department of Medical Oncology, Lung Unit, Institut Gustave Roussy, F-94805 Villejuif, France

**Keywords:** NSCLC, MAPK, EGFR, Ki-67

## Abstract

Activation of the ERK1/2 pathway is involved in malignant transformation both *in vitro* and *in vivo*. Little is known about the role of activated ERK1/2 in non-small cell lung cancer (NSCLC). The purpose of this study was to characterise the extent of the activation of ERK1/2 by immunohistochemistry in patients with NSCLC, and to determine the relationship of ERK1/2 activation with clinicopathological variables. Specimens from 111 patients with NSCLC (stages I–IV) were stained for P-ERK. Staining for epidermal growth factor receptor (EGFR) and Ki-67 was also performed. In all, 34% of the tumour specimens showed activation for ERK1/2, while normal lung epithelial tissue was consistently negative. There was a strong statistical correlation between nuclear and cytoplasmic P-ERK staining and advanced stages (*P*<0.05 and *P*<0.001, respectively), metastatic hilar or mediastinal lymph nodes (*P*<0.01, *P*<0.001), and higher T stages (*P*<0.01, *P*<0.001). We did not find correlation of nuclear or cytoplasmic P-ERK staining with either EGFR expression or Ki-67 expression. Total ERK1/2 expression was evaluated with a specific ERK1/2 antibody and showed that P-ERK staining was not due to ERK overexpression but rather to hyperactivation of ERK1/2. Patients with a positive P-ERK cytoplasmic staining had a significant lower survival (*P*<0.05). However, multivariate analysis did not show significant survival difference. Our study indicates that nuclear and cytoplasmic ERK1/2 activation positively correlates with stage, T and lymph node metastases, and thus, is associated with advanced and aggressive NSCLC tumours.

In the United States and Europe, lung cancer is the leading cause of cancer death. Non-small cell lung cancer (NSCLC) is the most common type, accounting for 75–80% of all lung cancers (Dy and Adjei, 2002a). In spite of important advances in surgery, radiation, chemotherapy, and the efforts involved in early detection and prevention, the survival of patients with NSCLC has not changed significantly over the past 20 years (Devesa *et al*, 1995). This is due to the fact that patients with early stage disease can be effectively treated with surgery, but most patients present at diagnosis with advanced stage, which is essentially incurable, since systemic chemotherapy has poor long-term results in these patients. Even after surgery, 50% of operated patients will develop metastatic disease (Dy and Adjei, 2002a). All these facts emphasise the need for new early detection tools and for more effective therapies. Our knowledge of the molecular events associated with the biology of NSCLC has substantially increased over the past decade, and extensive molecular and cytogenetic alterations have been characterised in these tumours (Fong *et al*, 1999). Many molecular mechanisms associated with the pathogenesis of lung cancer offer the exciting possibility of becoming new targets for lung cancer therapy (Dy and Adjei, 2002a, 2002b). A better understanding of the molecular mechanisms involved in lung carcinogenesis and in the progression of this disease is urgently needed to design effective treatments.

One of the signalling pathways that has been extensively studied *in vitro* is the RAS–RAF–MEK–ERK pathway. Most of the studies carried out to characterise this pathway have been conducted in fibroblasts, although more recently there is also an increasing number of studies in epithelial cell lines (Cobb, 1999). The extracellular signal regulated kinase (ERK1/2) also called the mitogen-activated protein kinase (MAPK), is activated by dual phosphorylation on both Thr202 and Tyr204 residues. This activation is mediated by activated MEK1, which in turn is phosphorylated by RAF-1, and RAF-1 is activated by RAS (for reviews see Cobb, 1999; Widmann *et al*, 1999; Chang and Karin, 2001). The only known substrates of MEK1 are ERK1 and ERK2, which makes MEK1 an important point to control ERK signalling, and a potential therapeutic target. Activated ERK1 and ERK2 can phosphorylate a variety of nuclear substrates such as the transcription factors Elk-1, Ets1 and Sap1a, and also cytosolic targets such as phospholipase A2, and S6 Kinase p90^rsk^ among others (Widmann *et al*, 1999). Growth factors such as EGF, HGF and PDGF, as well as a variety of other stimuli activate ERK1/2, and activation of this kinase has been frequently associated with proliferation or differentiation depending on several factors such as cellular context and the length of the activation (Widmann *et al*, 1999). It is also known that several oncogenes can constitutively activate ERK, and that this uncontrolled activation of ERK can lead to malignant transformation both *in vitro* and *in vivo* (Webb *et al*, 1998). Furthermore, activated ERK1/2 and/or increased levels of ERK1/2 have been reported in a variety of human tumour cell lines (Hoshino *et al*, 1999) and epithelial cancer tissues such as breast (Sivaraman *et al*, 1997; Adeyinka *et al*, 2002), kidney (Oka *et al*, 1995), colon (Sebolt-Leopold *et al*, 1999) and head and neck cancers (Albanell *et al*, 2001). More recently, Blackhall *et al* (2003) have shown P-ERK activation in small cell lung cancer (SCLC) of a high percentage of patients studied. Although there is some evidence of the presence of activated ERK1/2 in NSCLC primary cancer cells derived from tumours as compared to normal lung cells (Hoshino *et al*, 1999), the extent of this activation and its meaning and role in patients with NSCLC remain to be defined. Therefore, we analysed the expression of phosphorylated (activated) ERK1/2 by immunohistochemistry with a phosphospecific antibody in a nonselected patient population with histologically proven NSCLC, and made correlations of the expression of this kinase with different clinical parameters and with survival, in an attempt to understand the role of activated ERK1/2 in these patients.

## MATERIAL AND METHODS

### Tissue specimens and patient characteristics

All patients were treated at the University Hospital of the University of Navarra (Clínica Universitaria de Navarra, Pamplona, Spain) from December 1994 to June 2003. Paraffin-embedded tissue specimens from 111 patients were obtained according to institutional review board-approved protocols. These tissue specimens were obtained either from biopsies of the primary tumours corresponding to patients with advanced nonoperable NSCLC or from surgical specimens in those patients who were operated. In both cases, biopsy specimens were obtained prior to any chemo- or radiation therapy treatment. All the samples were fixed in 10% buffered formalin. Sections (5 *μ*m) from the paraffin tissue blocks were processed for immunohistochemical analysis. One section was stained with haematoxylin and eosin (H&E) and reviewed by at least two pathologists to confirm the presence of NSCLC in the specimen, and also for appropriate histopathological characterisation.

All patients had an ECOG performance status of 0 or 1, adequate baseline organ function (defined as a leucocyte count >3 × 10^9^ l^−1^ (absolute granulocyte count >1.5 × 10^9^ l^−1^), platelet count >75 × 10^9^ l^−1^, normal liver function tests and serum creatinine level <1.4 mg dl^−1^) and no other severe co-morbid conditions. A written signed informed consent was obtained from all patients before treatment.

Patient stage was determined according to the TNM staging system. Surgery was performed as the initial treatment for medically operable stages I, II and IIIA (non-N2 tumours). Lobectomy and ipsilateral mediastinal lymph node sampling was the treatment of choice. Postoperative radiotherapy was added depending on the pathologic findings. Specifically, radiation therapy was recommended with pathologically confirmed metastatic hilar or mediastinal lymph nodes. Postoperative radiotherapy consisted of 45 grays (Gy) using conventional fields and fractionation schedules.

Patients with locally advanced NSCLC were initially treated with chemotherapy. Intravenous infusions of paclitaxel 135 mg m^−2^ over 3 h and cisplatin 120 mg m^−2^ over 6 h were delivered on day 1. Either vinorelbine 30 mg m^−2^ or gemcitabine 800 mg m^−2^ over 30 min was administered on days 1 and 8. Cycles were repeated every 4 weeks. Four cycles were delivered to responding patients before administering radical radiotherapy with concurrent chemotherapy. Patients who did not respond to this treatment were administered radical hyperfractionated radiotherapy (69.6 Gy) soon after progression to chemotherapy was detected. Stage IV patients were treated with the chemotherapy schedule previously described for stage III patients. Median follow-up time of the whole group was 30 months (range 1–87).

Clinicopathological characteristics including age, gender, histology, stage and grade of differentiation were evaluated. We also evaluated the expression of the epidermal growth factor receptor (EGFR) and the nuclear antigen Ki-67 by immunohistochemistry in order to assess whether there was any correlation between activation of ERK1/2 and the expression of EGFR or the proliferation.

### Immunohistochemistry

Samples from 111 patients were studied by immunohistochemistry. Rabbit polyclonal antibodies specific against the dual phosphorylated form of ERK1/2 (Thr202/Tyr204) were used (Cell Signaling, Beverly, MA, USA) at a dilution of 1 : 100 (determined after preliminary serial dilution studies). Samples were incubated overnight at 4°C with the primary antibody. Immunocytochemical reaction was shown using the EnVision® TM intensifying kit (Dako, Carpinteria, CA, USA). Positive controls were ERK1/2-activated HTB58 human lung cancer cells fixed in 10% buffered formalin, which were analysed by immunohistochemistry with the anti-P-ERK polyclonal antibody. Activation of ERK1/2 was achieved by culturing the cells in a medium containing 20% fetal calf serum (FCS) after 16 h of serum deprivation (medium with 0.4% FCS) (data not shown). We also performed Western blot analyses of the induction of P-ERK after treatment of the cells with two known activating agents, fetal bovine serum (20%) and TPA (100 ng ml^−1^) (data not shown). Negative controls consisted of incubation of serial (positive) lung tumour sections with an unrelated IgG rabbit polyclonal antibody. The results of the staining were reviewed independently without knowledge of the clinicopathological data by three of the co-authors (SVC, CGC and LMM) who scored the percentage of positive nuclei and/or cytoplasms within tumours and in the normal matching lung at high magnification. Average of the scores was registered and no significant divergence was found in the scoring among the observers. Serial cuts (3 *μ*m) of a subset of the specimens (five samples that had high percentage of ERK1/2 activation and five samples that had no activated ERK1/2) were stained using a specific antibody against total ERK1/2 (Cell Signaling, Beverly, MA, USA). This experiment was also carried out using a dilution of 1 : 100 as described for the P-ERK antibody. The median percentage of P-ERK stained cells was 15% and this was chosen as the cutoff value. A score of 15% or higher was considered positive. Intensity of the staining was also registered and evaluated in a semiquantitative way but the intensity of the staining was fairly similar from specimen to specimen, and its inclusion in the statistical analysis did not modify the calculations based on extension alone; therefore, it will not be reported here.

The epidermal growth factor receptor and Ki-67 primary antibodies were from Novocastra (Newcatle, UK) and were used at 1 : 20 and 1 : 100 dilutions, respectively. They both were used according to the manufacturer's recommendations, including the use of appropriate positive and negative controls for each antibody. For the EGFR staining, three of the authors (GT, CGC and LMM) scored the specimens as positive, when any intensity and extension of staining was apparent, or negative when no signs of staining at all were seen, as described by other authors (Albanell *et al*, 2001). A positive score for the Ki-67 staining was considered when more than 5% of the tumour nuclei were stained as described before (Xu *et al*, 2002).

## STATISTICAL ANALYSIS

All statistical methods used were carried out using the statistical package SPSS Data Analysis Program version 9.0. The methods used were the Fisher's exact test, the *χ*^2^ test, the Mann–Whitney *U*-test, the Kaplan–Meier log rank test and the Cox regression test for univariate and multivariate survival analysis.

## RESULTS

A total of 111 patients with histologically proven NSCLC were evaluated in this study. The characteristics of these patients are shown in Table 1

**Table 1:** Characteristics of the patients

	**No. of patients (*n*=111)**	**% Total**
*Age*		
Median	62	
Range	31–87	
*Gender*		
Male	93	83.7
Female	18	16.3
*Histology*		
AC	50	46
SC	57	51
Mixt	3	3
*T*		
1	36	32.5
2	48	43.2
3	12	10.8
4	11	9.9
*x*	3	2.7
*Grade*		
1	9	13
2	28	40.6
3	32	46.4
*N*		
0	73	65.8
1	15	13.5
2	16	14.4
3	7	6.3
*Stage*		
I	62	55.9
II	18	16.2
III	28	25.2
IV	3	2.7

**Figure 1:**
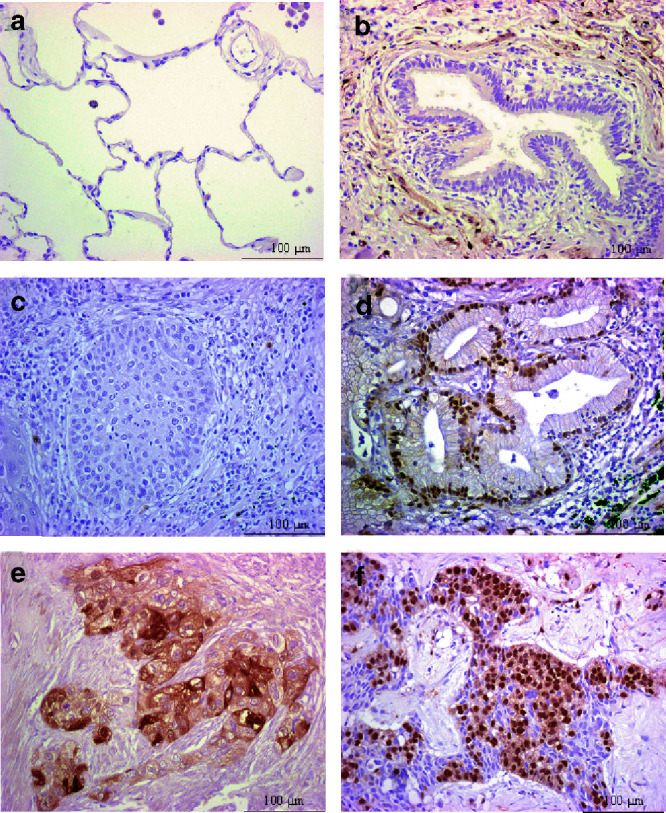
Immunohistochemical staining of normal lung and NSCLC specimens with a specific antibody against P-ERK: (**A**) normal alveolar epithelium, negative staining; (**B**) normal bronchiolar epithelium, negative staining; (**C**) squamous cell carcinoma, negative staining; (**D**) adenocarcinoma with nuclear P-ERK staining; (**E**) squamous cell carcinoma with cytoplasmic and moderate nuclear staining; (**F**) adenocarcinoma with nuclear and extensive cytoplasmic staining.

. The normal adjacent tissue in 30 of the samples was also studied. Normal lung tissue was negative for ERK1/2 activation in all the 30 samples studied. Specifically, type I and II alveolar pneumocytes did not show any staining for P-ERK, and bronchiolar epithelium was also negative for P-ERK (Figure 1A and B

). Endothelial cells were also negative for P-ERK staining in the histologically normal areas.

Immunohistochemical staining for P-ERK in the tumours showed that a variable percentage of cells was stained in each sample and that the staining could be located in the nucleus or in the cytoplasm, but most often it was present in both nucleus and cytoplasm, usually with a predominant staining in the latter. We recorded and analysed the staining for P-ERK according to its intracellular location. Examples of positive and negative staining are shown in Figure 1C–F. For statistical purpose, patients were arranged in two groups: (a) patients with less than 15% of tumour cells stained for P-ERK and (b) patients with 15% or more cells stained. Using this cutoff, specimens were also scored according to the location of the staining: nuclear staining (with or without accompanying cytoplasmic staining) or cytoplasmic staining (with or without accompanying nuclear staining). Although not reported, the results obtained shifting the cutoff values from 5–10 to 20–25% had a similar trend to those shown here using the cutoff value of 15%. Overall, 65.8% of tumors were negative and 34.2% positive when nuclear and cytoplasmic staining was considered. Among positive NSCLC, at least half of the tumours showed ERK1/2 activation preferentially located at the periphery of the tumour.

We found a strong statistical correlation between a positive nuclear and cytoplasmic P-ERK staining and advanced stages (*P*=0.04, *P*=0.001), higher T stages (T1–2 *vs* T3–4; *P*=0.005, *P*=0.001), and the presence of metastatic lymph nodes (N0 *vs* N1–3; *P*=0.001, *P*=0.001). However, there was no statistically significant difference between patients whose tumours had negative staining *vs* those patients whose tumours had positive staining either in the nucleus or in the cytoplasm when comparing age, sex and histology (squamous cell carcinoma *vs* adenocarcinoma) (Table 2

**Table 2:** Clinicopathological factors and their relationship to the expression of the different proteins assessed

	**P-ERK nuclear**	**P-ERK cytoplasmic**	**Ki-67**	**EGFR**
Age	*P*=0.7	*P*=0.115	*P*=0.277	*P*=0.143
Sex	*P*=0.349	*P*=1	*P*=0.058	*P*=0.146
Histology	*P*=0.812	*P*=0.65	***P*=0.04**	*P*=0.28
Grade	*P*=0.271	***P*=0.046**	*P*=0.653	*P*=0.54
Stage	***P*=0.04**	***P*=0.000**	*P*=0.902	*P*=0.3
T	***P*=0.005**	***P*=0.000**	*P*=0.586	*P*=0.58
N	***P*=0.001**	***P*=0.000**	*P*=0.897	*P*=0.64
EGFR	*P*=0.289	*P*=0.63	*P*=0.096	
Ki-67	*P*=0.211	*P*=0.424		

Bold type values indicate significance at levels <0.05. *P*, probability value.

). The grade of differentiation was only associated with cytoplasmic P-ERK staining but not with nuclear staining (1 and 2 *vs* 3, *P*=0.046 for cytoplasmic staining and *P*=0.271 for nuclear staining).

Staining with a specific total ERK1/2 antibody of a subset of 10 tumour specimens (five negative and five positive for P-ERK staining) showed the presence of a homogeneous staining for total ERK1/2 in the 10 specimens independently of their P-ERK status. These results suggest that the expression of P-ERK is not the consequence of overexpression of ERK1/2 but rather the result of hyperactivation of ERK1/2 in the specimens.

Trying to understand whether there is any relationship between the presence of activated ERK1/2 and the EGF-dependent growth regulatory pathway in NSCLC, staining of the same samples for EGFR was carried out. In some systems, activated ERK1/2 correlates well with proliferation. Thus, immunohistochemical staining for Ki-67 (as a surrogate marker of cell proliferation) was also performed in this series of tumours. Neither EGFR nor Ki-67 expression correlates in the specimens analysed with activated ERK1/2 (cytoplasmic or nuclear) as detected by immunohistochemistry (Table 2).

**Figure 2:**
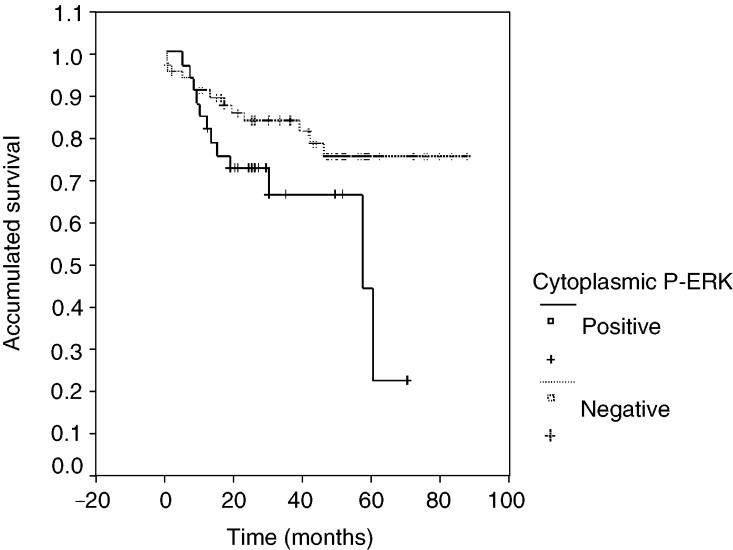
Kaplan–Meier survival plot by cytoplasmic P-ERK expression (15% cutoff). Survival plot of all patients.

Univariate analysis of clinicopathological factors relevant to patient survival showed that the following factors studied were statistically significant for the survival of patients: stage (*P*=0.001), T (*P*=0.001), N (*P*=0.001) and a positive cytoplasmic P-ERK staining (*P*=0.03). Interestingly, the presence of nuclear P-ERK staining was not statistically significant (*P*=0.509). Figure 2

shows a Kaplan–Meier plot of patients whose tumours had positive cytoplasmic staining (15% or more cells with immunostained cytoplasm) and those whose tumours had negative P-ERK staining (less than 15% of cells with immunostained cytoplasm). This plot shows that the median survival of patients with positive P-ERK staining is 57 months (95% confidence interval 11–102 months), whereas for patients with negative staining the median survival has not been reached, and this difference is statistically significant with *P*=0.02. The estimated 5-year survival rate was 43% for patients with positive P-ERK staining *vs* 77% for patients whose tumours had negative staining for P-ERK. However, survival analysis of the NSCLC patients analysed in this study did not show any significant differences according to the expression of either EGFR (*P*=0.18) or Ki-67 (*P*=0.13).

In order to determine whether P-ERK staining is an independent prognostic factor in NSCLC, as it is strongly associated with advanced stages and metastatic tumours, we performed a multivariate analysis by adjusting phospho-ERK1/2 activation to clinical stage (a parameter that depends on T and N), which influences patient survival. The test showed that patients with negative P-ERK did not live significantly longer than patients with positive P-ERK (*P*=0.69).

## DISCUSSION

Although an increased amount of information has been gathered on the role of the activation of ERK1/2 in a variety of cell lines, very little is known about its potential role in human cancer. A few previous reports tried to address this issue by studying the degree of activation of ERK1/2 in different human tumours. Oka *et al* (1995) showed that there was a constitutive activation of ERK1/2 in 48% of renal cancer samples (12 out of 25 patients) when compared to normal kidney tissue, suggesting that this signalling pathway could play a role in renal cancer. It was also reported that activated ERK1/2 was present in 58% of hepatocellular carcinomas studied, and that this activation was higher than in adjacent noncancerous lesions (Ito *et al*, 1998). Another report found no ERK1/2 activation in normal prostatic tissues and an increased expression of activated ERK1/2 in prostatic carcinoma tissues in advanced stages and in recurrent cases (Gioeli *et al*, 1999). There was also evidence of activated ERK1/2 in patients with glioma (Mandell *et al*, 1998), breast cancer (Sivaraman *et al*, 1997; Adeyinka *et al*, 2002), head and neck cancer (Albanell *et al*, 2001) and melanoma (Cohen *et al*, 2002). In our study, we show for the first time that normal matching lung tissue in the tumour specimens analysed, as previously reported for other tumour types, does not show activated ERK1/2, while activation of ERK1/2 is frequently found in NSCLC. Little evidence existed regarding the expression of activated ERK1/2 in lung cancer and, specially, in NSCLC. A previous report showed increased levels of activated ERK1/2 in a very reduced number of lung cancer tissues when compared to matching normal lung (Hoshino *et al*, 1999). Recently, Blackhall *et al* (2003) described that ERK1/2 is activated in SCLC, and Mukohara *et al* (2003) have studied some elements of the EGFR signalling pathway in a much smaller number of NSCLC patients. We have also found that a high percentage of NSCLC patients (38 out of 111) present ERK1/2 activation. In this regard, we have seen that a high percentage of positive NSCLC specimens (50%) presented ERK1/2 activation at the periphery of the tumour. This pattern of staining had been described by Albanell *et al* (2001) in head and neck cancers and also by Mukohara *et al* (2003) in NSCLC. Therefore, it seems that activated ERK1/2 is a landmark of a subset of NSCLC.

In the present work, we used phosphospecific antibodies to identify activated ERK1/2. The antibody used in this study is the same antibody used in most of the immunohistochemistry studies performed to date (Sivaraman *et al*, 1997; Mandell *et al*, 1998; Albanell *et al*, 2001; Adeyinka *et al*, 2002; Cohen *et al*, 2002). Besides, this antibody was validated in our lab specifically for formaline-fixed tissues (data not shown). There are other methods to assess the phospho-ERK status in patient specimens, such as Western blot. This technique has the inconvenience of requiring a larger amount of tissue, and that the inclusion of adjacent and stromal tissues along with the tumour in the extracts may mask the real activation status of ERK1/2.

To our knowledge, only a few studies published to date provide data on the clinical outcome of patients with activated ERK1/2. In breast cancer patients, phosphorylated ERK1/2 (as determined by immunohistochemistry) may predict a poor response to hormonal therapy and, in a subset of patients, a worse survival (Mueller *et al*, 2000; Gee *et al*, 2001). In SCLC, and regarding cytoplasmic staining of phospho-ERK1/2, the activation of this MAPK positively correlates with survival. In our work, we have seen a strong correlation of activated ERK1/2 with some clinicopathological variables. In particular, we have demonstrated that there is a strong correlation between positive nuclear and cytoplasmic staining for P-ERK and more aggressive tumours (i.e. advanced stage tumours (III and IV), tumours with hilar or mediastinal lymph node metastases, and tumours with higher T stage), and also between cytoplasmic staining and the grade of differentiation. With regard to survival, although we have seen in invariable analysis that P-ERK cytoplasmic expression may predict poor survival, multivariate analysis did not confirm that cytoplasmic ERK activation could be used as an independent prognostic factor. This may be due to the strong association of P-ERK with advanced stage tumours, metastatic tumours and tumours with higher T stage. In NSCLC, a recent report on the expression of EGFR and downstream-activated signalling factors activated by this receptor (Mukohara *et al*, 2003) showed that P-ERK is activated in about one-third of NSCLC, as we report in the present study in a much larger series. In contrast to our data, they found no correlation of the activation of this MAPK with clinicopathological variables. Another difference in our results with respect to those published by Mukohara *et al* (2003) is that we have almost the double number of patients (*n*=111 *vs N*=60). This gives more statistical support and confidence to the results obtained in this study. Besides, we have used an antibody that is different from the one used by Mukohara's group. We have validated the specificity of the antibody by specific experiments and by its utilisation in other studies by previous authors (Sivaraman *et al*, 1997; Mandell *et al*, 1998; Albanell *et al*, 2001; Adeyinka *et al*, 2002; Cohen *et al*, 2002). These two differences may explain the divergent results between our studies in relation to phospho-ERK and its correlation with clinicopathological variables.

A previous report showed that in head and neck squamous cancer, activated ERK1/2 correlated with the expression of the EGFR (Albanell *et al*, 2001). In our patients we also looked at the correlation between EGFR and the expression of activated ERK1/2, but we did not find such a correlation. It is quite likely that activation of the EGFR, rather than merely its expression, is what is relevant in terms of activation of ERK. If this is the case, it could also be of interest to determine the levels of phospho-EGFR in addition to the levels of activated ERK1/2 in patients with NSCLC to test whether patients with a positive staining could benefit from specific inhibitors of EGFR, like Iressa. In fact, both the phosphorylated EGFR and P-ERK specific antibodies have already been used to study the pharmacodynamic effects of Iressa in skin biopsies of patients treated with the drug (Albanell *et al*, 2002). Our results may suggest a greater involvement of other growth factors in the activation of the ERK1/2 signalling pathway and, therefore, in the progression of NSCLC. As an example, a potential role of FGF in NSCLC has already been reported (Berger *et al*, 1999; Guddo *et al*, 1999).

Our data also suggest the relevance of the intracellular location of the immunostaining for P-ERK. In SCLC, Blackhall *et al* (2003) had reported that cytoplasmic and not nuclear phospho-ERK staining correlated with patients survival. In our work, we have shown that only the cytoplasmic staining was associated with a poorer survival in NSCLC. As ERK1/2 has both nuclear and cytoplasmic substrates (Widmann *et al*, 1999), this observation could indicate an important role of cytoplasmic substrates of ERK1/2 in advanced and metastatic tumours. Recently, more cytoplasmic substrates of phospho-ERK1/2 involved in apoptosis (such as caspase 9) have been described, indicating that ERK1/2 activation directly regulates programmed cell death (Allan *et al*, 2003). In lung cancer, although there is some indication of the possible contribution of some of the nuclear substrates of ERK1/2 to the biology and prognosis of NSCLC patients (Levin *et al*, 1995; Volm *et al*, 2002), very little is known about the possible role of cytoplasmic substrates of ERK1/2 in NSCLC.

Another potential implication of our findings is related to a new class of therapeutic agents: the MEK1 inhibitors. Although inhibitors of MEK1, like PD98059 (Alessi *et al*, 1995; Dudley *et al*, 1995) and U0126 (Duncia *et al*, 1998), have been used for quite a while in laboratory studies, it was not until recently that the first compound of this new class of drugs, called PD184352 (Sebolt-Leopold *et al*, 1999), entered clinical trials. Previous MEK1 inhibitors lacked solubility, which prevented their use in the clinical setting. Preliminary results of phase I trials with PD184352 have shown encouraging results, and a clinical trial of this agent in patients with NSCLC has already started (Dy and Adjei, 2002a). Monitoring the P-ERK status in biopsies of NSCLC patients entering clinical trials of the newly introduced inhibitors of MEK1 will help to assess whether an activated ERK1/2 pathway is necessary for a MEK1 inhibitor to be effective, a question that at present remains unanswered.

In summary, we have shown that the detection of immunoreactivity for P-ERK in patients with NSCLC is associated with advanced and aggressive tumours. Our data also suggest that the analysis of ERK1/2 activation may be useful to identify a subgroup of patients with a poorer prognosis. Finally, our results suggest that the relevance of P-ERK might not be related to the proliferation rate of NSCLC but rather to other tumour characteristics such as the resistance to undergo apoptosis. In this regard, the role of ERK1/2 in cell survival and regulation of apoptosis is well established in various systems (Xia *et al*, 1995; Mackeigan *et al*, 2000). Besides, in lung cancer, Brognard and Dennis (2002) have pointed out the importance of ERK1/2 activation in NSCLC cell lines, where it promotes cell survival and chemotherapeutic resistance. Further studies are warranted to explore the role of activated ERK1/2 in patients with NSCLC.
